# Comparative Effectiveness of Laparoscopic Sleeve Gastrectomy in Morbidly Obese and Super Obese Patients

**DOI:** 10.7759/cureus.20767

**Published:** 2021-12-27

**Authors:** Ishfaq A Khan, Ayaz A K, Muhammad Asghar, Kiran Abbas

**Affiliations:** 1 Department of Surgery, Barnsley Hospital, Barnsley, GBR; 2 Department of Surgery, Saqr Hospital, Ras al Khaimah, ARE; 3 Department of Surgery, Dr. Sulaiman Al-Habib Hospital, Dubai Healthcare City, ARE; 4 Department of Medicine, Jinnah Postgraduate Medical Centre, Karachi, PAK

**Keywords:** super obese, obese, morbidly obese, laparoscopic sleeve gastrectomy, effectiveness

## Abstract

Background

Laparoscopic sleeve gastrectomy (LSG) is a modified procedure derived from a biliopancreatic diversion (BPD)-duodenal switch. The present study evaluated the role of LSG in morbidly and super obese patients and compare its efficacy between the two groups.

Methodology

A retrospective review was conducted in Dr. Sulaiman Al Habib Specialist Hospital, Riyadh, KSA, from January 2020 to April 2021. Patients' records were divided into two groups, morbidly obese (body mass index (BMI): 40-49 kg/m^2^) and super obese (BMI: 50-59 kg/m^2^), who were admitted to the department for laparoscopic sleeve gastrectomy during the study duration. However, patients with a history of gut surgery, hernias, comorbid use of illicit substances, and psychiatric disorders were excluded. For all patients, a routine preoperative investigation protocol was conducted. Postoperative surgical complications were also recorded. The Clavien-Dindo classification (CDC) score was applied to record surgical complications. Data collection was done using a semi-structured questionnaire. The Statistical Package for Social Sciences (SPSS) version 26 (IBM, Chicago, USA) was used to perform data analysis.

Results

A total of 176 patient records were included in this analysis, of which 126 (71.6%) were females. There were 101 (57.1%) patients who were morbidly obese and 76 (42.9%) who were super obese. The mean duration of follow-up records in this study was 23.2 ± 3.6 weeks, which was slightly longer in the morbidly obese group. Change in BMI was higher in the super obese patients (18.6 ± 3.1 versus 10.5 ± 1.9). Final body weight was still lower in the morbidly obese group as they were relatively slimmer even before the procedure. A higher reduction in excess weight loss (EWL) is seen in the morbidly obese group. Comorbidity resolution status was also remarkable with the procedure. Overall, there were procedure-associated complications in 11 (10.9%) patients in the morbidly obese group and 10 (13.2%) in the super obese group.

Conclusion

Laparoscopic sleeve gastrectomy is a safe procedure in morbidly and super obese patients. It is effective in sustainable total and excess weight loss over time. It is also effective in comorbidity resolution. Complications with LSG are minimal and nonserious. LSG should be the recommended procedure in morbidly and super obese patients with adverse health consequences to improve their morbidity, mortality, and overall quality of life.

## Introduction

Over the last two decades, with changes in population characteristics, urbanization, calorie-dense food choices, and digitalization, as many other disease dynamics have evolved, so has obesity. Most noncommunicable illnesses are a result of obesity, but obesity itself has also been established as a preventable, chronic, and noncommunicable disease [1]. According to the World Health Organization (WHO), 13% of all adult individuals, more than 650 million, were obese globally in 2016 [2]. The health consequences of obesity are diverse, spanning from physical to psychological impacts.

Metabolic syndrome, diabetes mellitus (DM), coronary artery disease (CAD) and hypertension (HTN), breast and gastrointestinal (GI) cancers, osteoarthritis, depression, anxiety, and eating disorders all contribute to healthy years lost [3]. With the rapidly expanding statistics of obesity, it has been termed a pandemic. Medical advancements in managing obesity are also opening out. There is great research at the basic sciences and clinical level to standardize medical and surgical management of obesity. Sleeve gastrectomy is among such highly sophisticated surgical procedures that have shown great results in morbidly obese (body mass index (BMI): ≥40 kg/m^2^), super obese (BMI: ≥50 kg/m^2^), and super-super obese (BMI: ≥60 kg/m^2^) patients [4]. Other procedures include Roux-en-Y gastric bypass, biliopancreatic diversion (BPD) with duodenal switch, and adjustable gastric banding. Almost all procedures are now performed laparoscopically [5]. All such bariatric procedures are indicated only when dietary management, lifestyle modifications, and medical management fail.

Laparoscopic sleeve gastrectomy (LSG) is a modified procedure derived from a biliopancreatic diversion-duodenal switch. LSG is becoming a successful and popular treatment modality for morbidly obese patients to control their health-related consequences. The procedure has shown a greater impact on sustained weight loss in the long term. It is a relatively safe procedure with limited complications, shorter hospital time, and no foreign body implantation, and it does not require any gut anastomosis [6]. Literature has shown some great results of LSG with morbidly and super obese patients [4,6,7]; however, most literature from Pakistan is incomplete, such that obesity categories are not well defined and inclusion criteria are not robust [8-10]. Hence, this study was initiated to assess the role of LSG in morbidly and super obese patients and compare its efficacy between the two groups.

## Materials and methods

A retrospective review was conducted in Dr. Sulaiman Al Habib Specialist Hospital, Riyadh, KSA, from January 2020 to April 2021. The study was initiated after obtaining approval from the Institutional Review Board (reference #IRB-HMG-4665). All patients were included after obtaining informed consent.

Patients' records were divided into two groups, morbidly obese (BMI: 40-49 kg/m^2^) and super obese (BMI: 50-59 kg/m^2^), who were admitted to the department for laparoscopic sleeve gastrectomy during the study duration. Beyond the scope of this study, the patients were already informed about the risks and benefits associated with the procedures, possible complications, and outcomes. All patients had already consented to the procedure. However, patients with a history of gut surgery, hernias, comorbid use of illicit substances, and psychiatric disorders were excluded.

For all patients, a routine preoperative investigation protocol was conducted. It included full blood picture, electrolytes, lipid profile, fasting blood sugar, thyroid profile, prothrombin time, and partial thromboplastin time. Imaging conducted for these patients included chest X-ray, whole abdominal ultrasound, and upper gastrointestinal endoscopy. Cardiac safety was assessed for patients with CAD and/or HTN. Preoperative general anesthesia fitness was assessed using the American Society of Anesthesiologists (ASA) score [11].

The surgery was performed by a surgeon with experience of over eight years. The laparoscopic sleeve gastrectomy involved excising the stomach along the greater curvature. During the procedure, all aseptic measures were taken, and a 12-mm trocar was inserted on the supraumbilical lateral border of the right rectus. Several 5-mm trocars were then placed at the subxiphoid, right costal margin, and left costal margin and finally on the lateral border of the left rectus. The camera was inserted in the midline. For local anesthesia, bupivacaine was administered. The vascular supply of the greater curvature was divided using the harmonic scalpel extending from the left crus of the diaphragm to the distal part of the pylorus. By the end of the procedure, at least 80% of the stomach was removed, and a gastric sleeve (tube) of less than 60 cc was left. All perioperative findings were documented.

Postoperative surgical complications were also recorded. The Clavien-Dindo classification (CDC) score was applied to record surgical complications. It grades the severity of a complication on the basis of the type of intervention needed to manage that complication [12]. Data collection was done using a semi-structured questionnaire. It consisted of the sociodemographic profile of the patients, their medical and surgical comorbidity status, health consequences of obesity, obesity category (morbid/super), preoperative investigation profile, and weight assessment. Weight was assessed preoperatively, seven days postoperatively, one month postoperatively, and six months postoperatively. Excess weight loss (EWL) was calculated using the following formula [4]:

([Preoperative weight − Follow-up weight] / [Preoperative weight − Ideal body weight]) × 100

The ideal body weight was calculated assuming an ideal BMI (25 kg/m^2^).

All data were cleaned, entered, and analyzed using the Statistical Package for Social Sciences (SPSS) version 26 (IBM, Chicago, USA). Continuous data were presented as means and standard deviations (SDs). Categorical data were presented as frequencies and percentages. Post-stratification correlation tests were performed. P-value ≤ 0.05 was taken as significant.

## Results

A total of 176 patient records were included in this analysis, of which 126 (71.6%) were females. There were 101 (57.1%) patients who were morbidly obese and 76 (42.9%) who were super obese. Their baseline characteristics, preoperative weight, comorbidity status, and ASA score are classified in Table 1.

**Table 1:** Patient sociodemographic and clinical characteristics ASA: American Society of Anesthesiologists

Characteristics	Morbidly Obese Group	Super Obese Group
Number of cases	101 (57.1%)	76 (42.9%)
Age (years)	42.1 ± 13.3	38.7 ± 11.9
Gender		
Female	72 (71.3%)	54 (71.1%)
Male	29 (28.7%)	22 (28.9%)
Body mass index (kg/m^2^) baseline	42.9 ± 2.6	57.1 ± 3.1
Total bodyweight	119.3 ± 14.9	156.4 ± 17.3
ASA classification		
I	39 (38.6%)	26 (34.2%)
II	33 (32.7%)	26 (34.2%)
III	29 (28.7%)	23 (30.3%)
Comorbidities		
Type 2 diabetes mellitus	40 (39.6%)	32 (42.1%)
Hypertension	39 (38.6%)	34 (44.7%)
Dyslipidemia	30 (29.7%)	25 (32.9%)
Joint diseases	11 (10.9%)	9 (11.8%)

The mean duration of follow-up records in this study was 23.2 ± 3.6 weeks, which was slightly longer in the morbidly obese group. As shown in Table 2, weight changes were better in the super obese patients. Change in BMI was higher in the super obese patients (18.6 ± 3.1 versus 10.5 ± 1.9). Final body weight was still lower in the morbidly obese group as they were relatively slimmer even before the procedure. A higher reduction in excess weight loss (EWL) is seen in the morbidly obese group. All other weight-changing characteristics are shown in Table 2.

**Table 2:** Weight changes in the morbidly obese and super obese patients over time BMI: body mass index TWL: total weight loss EWL: excess weight loss

Parameters	Morbidly Obese Group	Super Obese Group
Follow-up duration (weeks)	22.6 ± 4.9	21.1 ± 4.7
Change in BMI	10.5 ± 1.9	18.6 ± 3.1
Final body weight (kg)	81.5 ± 11.4	104.1 ± 12.7
Total body weight loss (kg)	37.8 ± 10.1	52.3 ± 10.2
TWL%	31.7 ± 6.6	33.4 ± 4.1
EWL%	62.3 ± 10.8	59.1 ± 7.2

Comorbidity resolution status was also remarkable with the procedure. It was comparable for both groups as shown in Table 3. The highest improvement was seen in dyslipidemia in both study groups (84% and 86.6%), and the least improvement was seen in joint diseases (66% in each group) (Table 3).

**Table 3:** Comparison of comorbidity resolution in the morbidly obese and super obese groups

Comorbidity Resolution		Morbidly Obese Group	Super Obese Group
Type 2 diabetes mellitus		33 (82.5%)	25 (78.1%)
Hypertension		30 (76.9%)	27 (79.4%)
Dyslipidemia		26 (86.6%)	21 (84%)
Joint diseases		7 (66%)	6 (66.6%)

Overall, there were procedure-associated complications in 11 (10.9%) patients in the morbidly obese group and 10 (13.2%) in the super obese group. Surgical site infection and staple line leak/bleeding were similar in both groups. One super obese patient developed deep vein thrombosis. On CDC, all complications were mild to moderate (Table 4).

**Table 4:** Comparison of comorbidity resolution in the morbidly obese and super obese groups

Complications	Morbidly Obese Group	Super Obese Group
Total complications	11 (10.9%)	10 (13.2%)
Urinary tract infection	2 (2%)	1 (1.3%)
Nausea or vomiting	4 (4%)	3 (3.9%)
Deep vein thrombosis	0 (0%)	1 (1.3%)
Surgical site infection	1 (1%)	1 (1.3%)
Staple line bleeding	1 (1%)	1 (1.3%)
Staple line leak	2 (2%)	1 (1.3%)
Clavien–Dindo classification score		
1	7 (6.93%)	7 (9.21%)
2	4 (3.96%)	4 (5.26%)
3a	2 (1.98%)	1 (1.32%)
3b	-	-
4	-	-
5	-	-

## Discussion

Surgical management of obesity is the most effective intervention for weight loss and management of obesity-associated adverse health consequences. Weight loss surgery is widely accepted in the medical community for rapid and durable weight loss in morbidly obese patients. It not only improves the overall survival rate but also protects against comorbidities associated with obesity - CAD, HTN, DM, obstructive sleep apnea, and malignancies [13].

Laparoscopic sleeve gastrectomy is a minimally invasive, safe, and effective procedure in morbidly obese and super obese individuals with obesity-associated health consequences. The results of this study support the procedure of LSG as the primary surgical intervention for obesity. In this study, the results were remarkable for both morbidly and super obese patients. EWL was greater in morbidly obese; however, reduction in BMI was higher in super obese patients.

The results of other studies are also comparable. The work of Ece et al. concludes LSG as a safe and feasible stand-alone bariatric surgical procedure for the management of morbidly and super obese patients [4]. In our study, TWL and EWL were good with LSG in both groups, with greater TWL% in the super obese group and greater EWL% in the morbidly obese group. The results of a recently published systematic analysis showed that the maximum EWL for LSG occurred 24-36 months postoperatively, which was 64%-66% [14]. However, the sample was not followed for that long duration in our study. In another study, which compared the extent of weight loss with LSG in diabetic and nondiabetic groups, the mean EWL% at six months was 42.18% for the diabetic group and 61.22% for the nondiabetic group [15].

The main aim of any bariatric procedure is to improve adverse health consequences and comorbidities associated with obesity. In patients with morbid obesity, diabetes mellitus, cardiovascular diseases, and malignancies remain the culprit of morbidity and mortality [16,17]. The results of this study have shown great improvement in the diabetic and cardiovascular status of patients after the LSG procedure. Similarly, the work of Omana et al. also showed 100% resolution of DM, 78% resolution of HTN, and 87% reduction in hyperlipidemia with LSG. Their results with LSG were also significantly better than other bariatric procedures [18].

LSG is a safe procedure with minimal postoperative complications. Also in terms of long-term nutritional deficiency, LSG has been regarded as safe as compared to other procedures such as biliopancreatic diversion (BPD) [19]. In our study, 10%-13% of patients experienced some postoperative complications, although none was serious or life-threatening. Other bariatric procedures are reportedly likely to complicate severe diarrhea, steatorrhea, anastomotic ulcer, hypoalbuminemia, persistent vomiting, and severe anemia [4].

## Conclusions

Laparoscopic sleeve gastrectomy (LSG) is a safe procedure in morbidly and super obese patients. It is effective in sustainable total and excess weight loss over time. It is also effective in comorbidity resolution. Complications with LSG are minimal and nonserious. LSG should be the recommended procedure in morbidly and super obese patients with adverse health consequences to improve their morbidity, mortality, and overall quality of life.
